# GH Mediates Exercise-Dependent Activation of SVZ Neural Precursor Cells in Aged Mice

**DOI:** 10.1371/journal.pone.0049912

**Published:** 2012-11-27

**Authors:** Daniel G. Blackmore, Jana Vukovic, Michael J. Waters, Perry F. Bartlett

**Affiliations:** 1 Queensland Brain Institute, The University of Queensland, Brisbane, Queensland, Australia; 2 Institute for Molecular Bioscience, The University of Queensland, Brisbane, Queensland, Australia; National Institutes of Health, United States of America

## Abstract

Here we demonstrate, both *in vivo* and *in vitro*, that growth hormone (GH) mediates precursor cell activation in the subventricular zone (SVZ) of the aged (12-month-old) brain following exercise, and that GH signaling stimulates precursor activation to a similar extent to exercise. Our results reveal that both addition of GH in culture and direct intracerebroventricular infusion of GH stimulate neural precursor cells in the aged brain. In contrast, no increase in neurosphere numbers was observed in GH receptor null animals following exercise. Continuous infusion of a GH antagonist into the lateral ventricle of wild-type animals completely abolished the exercise-induced increase in neural precursor cell number. Given that the aged brain does not recover well after injury, we investigated the direct effect of exercise and GH on neural precursor cell activation following irradiation. This revealed that physical exercise as well as infusion of GH promoted repopulation of neural precursor cells in irradiated aged animals. Conversely, infusion of a GH antagonist during exercise prevented recovery of precursor cells in the SVZ following irradiation.

## Introduction

The subventricular zone (SVZ) of the lateral ventricle and the dentate gyrus of the hippocampus are the two niches in the brain where neurogenesis occurs throughout adulthood. The production of new neural cells has been shown to decline with age in both the SVZ [1], [2] and the hippocampus [3]. Voluntary exercise has been reported to stimulate neurogenesis [4], [5] and counteract the natural decline in neural precursor number [6], [7] within the hippocampus of aged animals. Although hippocampal neurogenesis has been shown to occur post-exercise, this appears not to be the case for the SVZ [8]. Using the neurosphere assay [9], however, we have previously observed increases in precursor cell activation within the SVZ of the aged brain after physical exercise [2]. Despite numerous studies the mechanism by which this neural precursor activation occurs is still to be fully elucidated.

In a previous study, we observed that neural precursor numbers did not increase in response to exercise in adult (6-month-old) growth hormone receptor null (GHR^−/−^) mice [2], suggesting a role for GH signaling in exercise-mediated precursor activation. In support of this notion, exercise has also been reported to trigger increases in GH levels [10]–[13].

Originally, GH was understood to be primarily involved in longitudinal growth, however, several studies have since reported that it can play a number of different roles depending on the system in which it is acting. In the adult mouse brain, functional GHR is present on resident neural stem and progenitor cells [14] and GH can positively regulate the activity of these cells [15], [16]. In a previous study, we demonstrated that neural precursor number in adult wild-type mice is positively affected by direct intracerebroventricular infusion of GH [14]. In addition, progressive loss of activated precursors and neurons has been shown to correlate with an age-dependent decline in GH secretion in both rodents and humans [17]. Together these findings suggest that GH signaling plays a critical role in the maintenance of precursor cell activity.

In this study, we have extended these lines of investigation in three key ways. Firstly, we have directly addressed whether GH mediates the exercise-induced increase in precursor activation in the aged brain. Secondly, we have examined whether the administration of GH counters the natural age-related decline in neural precursor cell number in the SVZ. Finally, we have assessed whether an even more extensive loss of precursor cells, induced by irradiation, can be rescued by the administration of GH.

**Figure 1:**
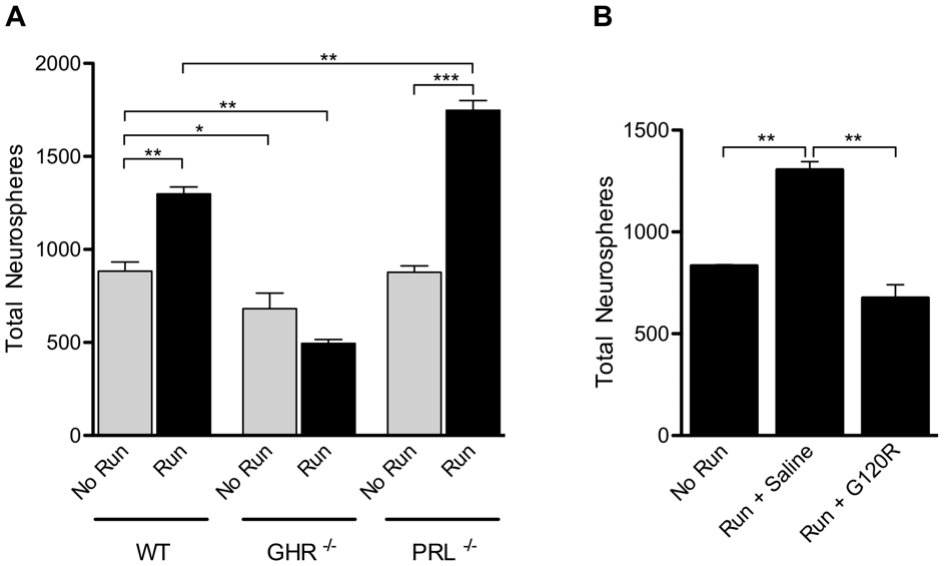
GH mediates exercise-induced activation of neural precursor cells in the aged SVZ. (**A**) After 21 days of exercise (“Run”), 12-month-old WT animals showed a significant increase in SVZ neurosphere number compared to age-matched non-exercised (“No Run”) WT animals (n = 5; p<0.01). Significantly fewer neurospheres were obtained from the SVZ of non-exercised GHR^−/−^ animals than non-exercised WT animals (n = 5; p<0.05), and no change was observed after exercise. Non-exercised PRL^−/−^ animals had similar neurosphere numbers to non-exercised WT animals (n = 4). After exercise, significantly more neurospheres were obtained from the SVZ of the PRL^−/−^ animals compared to both the non-exercised WT (p<0.01) and non-exercised PRL^−/−^ groups (n = 3; p<0.001). (**B**) *In vivo,* after 21 days of exercise, saline-treated animals (“Run + Saline”) showed a significant increase in neurosphere numbers compared to non-exercised animals (p<0.01). Infusion of G120R during exercise (“Run + G120R”) prevented an increase in neurosphere number, with levels in these animals comparable to the non-exercised group (p = 0.064). n = 3 for all treatments; one-way ANOVA with Bonferroni's multiple comparison post hoc test *p<0.05 **p<0.01 and ***p<0.001.

## Materials and Methods

### Animals

Adult female C57BL/6J mice, 12 months of age, were used throughout this study. GHR^−/−^ mice [18] were kindly provided by Professor Kopchick (Edison Biotechnology Institute) and PRL^−/−^ mice were originally provided by Associate Professor Ormandy (Garvan Institute of Medical Research, The University of New South Wales, Australia). Animals were genotyped by polymerase chain reaction (PCR) as previously described [19]. All animals were treated in accordance with the Australian Code of Practice for the Care and Use of Animals for Scientific Purposes. The University of Queensland Animal Ethics Committee approved all experiments under animal ethics approval number QBI/224/09. Control animals were placed three to a high-top cage (51×22×13 cm). Test animals were similarly housed but the cage was fitted with a single hanging running wheel (Able Scientific). All animals, both treated and untreated, ran between 2.6 and 4.1 km/day. No significant difference in total running distance was observed between treatment groups. All surgery was performed under ketamine/xylazine anaesthesia. All efforts were made to minimise pain and suffering (see Section “*Intracerebroventricular infusion*” for details of surgical technique).

**Figure 2:**
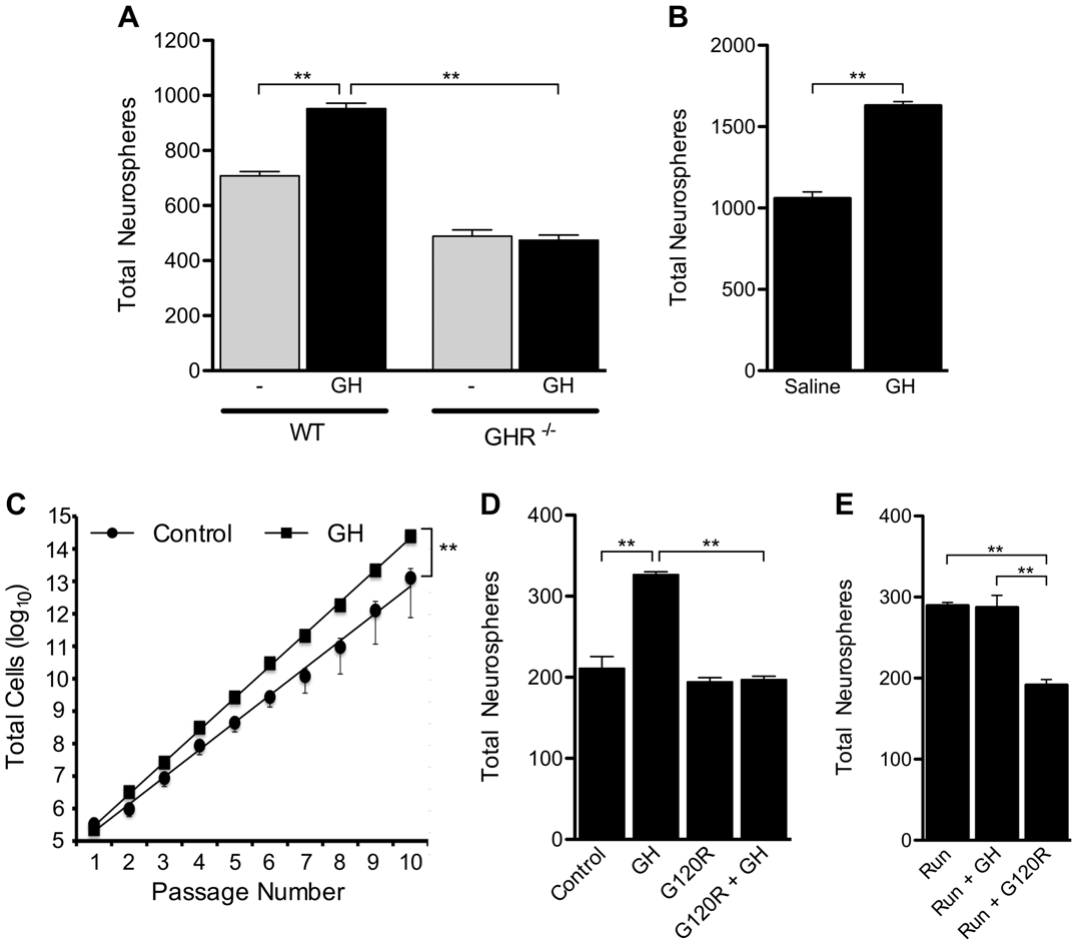
GH addition activates neural precursor cells derived from aged animals *in vitro* and *in vivo*. (**A**) *In vitro*, addition of GH to primary cells from WT animals resulted in a significant increase in neurosphere number compared to untreated WT cells (p<0.01). When compared to untreated GHR^−/−^ cells, those GHR^−/−^ cells that were treated with GH did not show an increase in total neurosphere number. (B) A 7 day *in vivo* intracerebroventricular infusion of GH resulted in significantly higher numbers of primary neurospheres compared to saline-treated animals (n = 2, Student t-test). (**C**) WT neural cells passaged over time with exogenous GH showed a significant increase in proliferation compared to control conditions (Linear regression analysis). (**D**) Primary cells from WT animals cultured in the presence of GH showed a significant increase in neurosphere number relative to control. Addition of the antagonist G120R alone had no significant effect on primary neurosphere numbers. Addition of the antagonist in the presence of GH resulted in no change in total neurosphere numbers compared to control but a significant decrease was seen relative to GH-treated cells. (**E**) Addition of GH *in vitro* to cells from exercised WT animals (“Run + GH”) did not increase neurosphere numbers when compared to exercised controls (“Run”). Addition of the GH antagonist G120R *in vitro* (“Run + G120R”) significantly decreased total neurosphere number relative to those obtained from exercised controls. n = 3 for all treatments; one-way ANOVA with Bonferroni's multiple comparison post hoc test unless otherwise stated. **p<0.01.

### Irradiation

For detailed methodology refer to Blackmore *et al.*, 2009. Briefly, mice were restrained within a plastic chamber and placed in a lead-shielded container (reducing exposure by 95%), leaving only the head exposed for irradiation. Single dose irradiation was induced by exposure to a Co^60^ source in a Gamma Cell 200 irradiator until a 3.5Gy dose had been given. After irradiation, animals were allowed to recover for two days before being allowed to exercise or having osmotic pumps inserted. Please see boxed sections in figures for schematics outlining experimental design.

**Figure 3:**
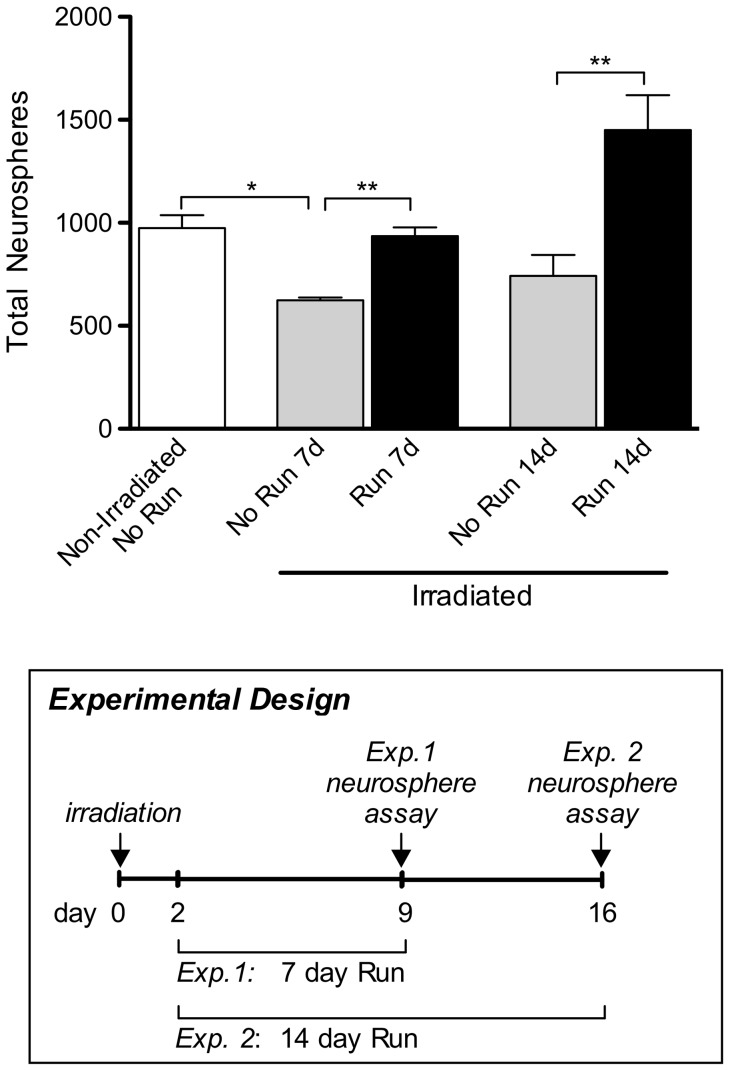
Physical exercise induces activation of neural precursor cells following irradiation. Neurosphere numbers were significantly lower from irradiated, sedentary mice (“Irradiated, No Run”) than from non-irradiated, sedentary (“Non-Irradiated, No Run”) animals after 7 days (p<0.05). Irradiated mice that had exercised (“Run 7d”) showed total neurosphere numbers comparable to those from non-irradiated, sedentary animals (n = 3). At both time points, neurosphere numbers from irradiated, exercised animals were increased significantly (p<0.01) relative to irradiated, sedentary animals (“No Run 7d” and “No Run 14d”). n = 6 for all experimental conditions, unless stated otherwise. Boxed panel shows schematic outlining experimental design. One-way ANOVA with Bonferroni's multiple comparison post hoc test. *p<0.05, **p<0.01.

### Primary neurosphere cultures

Mice were sacrificed by cervical dislocation, after which their brains were immediately removed and transferred to Petri dishes containing Hepes-buffered minimum essential medium (HEM), which consisted of minimum essential medium (Gibco/Invitrogen) supplemented with 16 mM HEPES (Sigma-Aldrich). Individual coronal sections at a thickness of 400 µm were collected between 2.54 and −0.34 mm relative to bregma. The 3 to 5 cell-layer-thick region of the SVZ was microdissected from each section using a fine scalpel. Tissue was finely diced and then enzymatically brought to a single-cell suspension using trypsin-EDTA (Gibco/Invitrogen) for 4 minutes at 37°C. The reaction was halted using trypsin inhibitor (Sigma-Aldrich) dissolved in HEM. Following centrifugation at 100 g for 5 minutes, cells were resuspended in 1 ml complete medium containing mouse Neurocult NSC Basal Medium plus Proliferation Supplement (Stem Cell Technologies) supplemented with 20 ng/ml epidermal growth factor (EGF; BD Biosciences), 10 ng/ml basic fibroblast growth factor (bFGF; Roche) and 20 ng/ml heparin (Sigma-Aldrich). Dependent upon experimental conditions, primary cultures were supplemented with receptor grade recombinant rat GH (50 ng/ml, Novozymes) and/or the competitive GH antagonist G120R (5 µg/ml, kind gift from Professor M. Waters). Neurospheres over 50 µm in diameter were counted 7 days after placement of cells in the assay. These were termed primary neurospheres.

**Figure 4:**
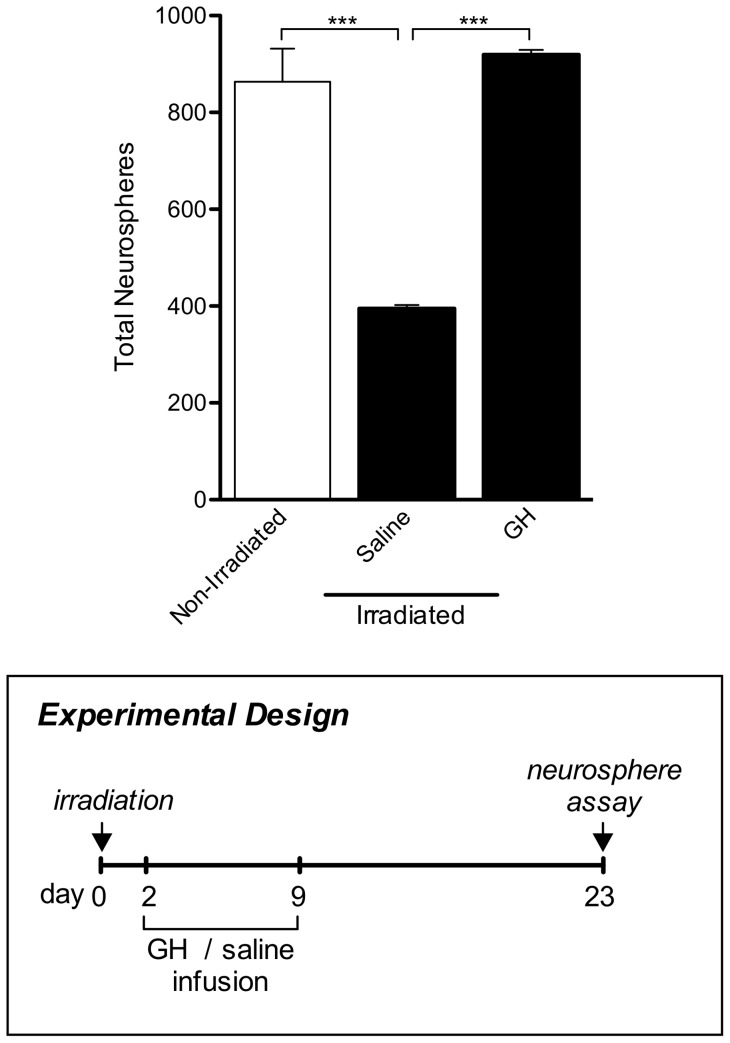
Direct GH infusion induces activation of neural precursor cells following irradiation. After a single 3.5Gy dose of irradiation, the total number of neurospheres was significantly reduced in saline-treated animals (“Saline”) compared to non-irradiated, untreated (“Non-Irradiated”) mice. When compared to saline-treated animals, infusion of GH (“GH”) resulted in significantly higher numbers of neurospheres 14 days post-infusion. Boxed panel shows schematic outlining experimental design. n = 3 for all experimental conditions. One-way ANOVA with Bonferroni's multiple comparison post hoc test. ***p<0.001.

### Intracerebroventricular infusion

The day before surgery, osmotic mini pumps at a flow rate of 0.5 µl/hour (Alzet #1007D, 7-day pump) or 0.11 µl/hour (Alzet #1004, 28-day pump) were loaded with recombinant rat GH (3 μg/ml; Novozymes Australia), GH antagonist G120R (400 µg/ml) or saline solution (0.9% sterile physiological saline), and attached to the infusion cannula. The entire apparatus was then stored overnight at 4°C in phosphate buffered saline. Three hours prior to surgery, pumps were incubated in a 37°C water-bath. Adult C57BL/6J mice were anesthetised via intraperitoneal injection using a ketamine/xylazine mixture (50 mg/kg and 8 mg/kg body weight, respectively). Once anesthetised, mice were mounted onto a stereotaxic frame and a 1.5 cm incision was made to expose the skull. A single hole was drilled in the skull (1.25 mm diameter) directly above the lateral ventricle (A/P: +0.2, M/L:±0.9, depth: −2.5 mm), and a 30-gauge cannula was lowered and fixed into place using cyanoacrylate adhesive, so as to enable infusion directly into the ventricle. The test mice received 36 ng of GH per day, or 252 ng over a 7-day period. Animals were sacrificed 10, 21 or 23 days following osmotic pump insertion. The GH antagonist, G120R was placed in the Alzet 28-day osmotic pump at a concentration of 400 µg/ml. The total concentration received was 1.056 µg/day, totalling 22.176 µg over the 21-day exercise period. Experimental design is schematically represented in boxed sections of the relevant figures.

**Figure 5:**
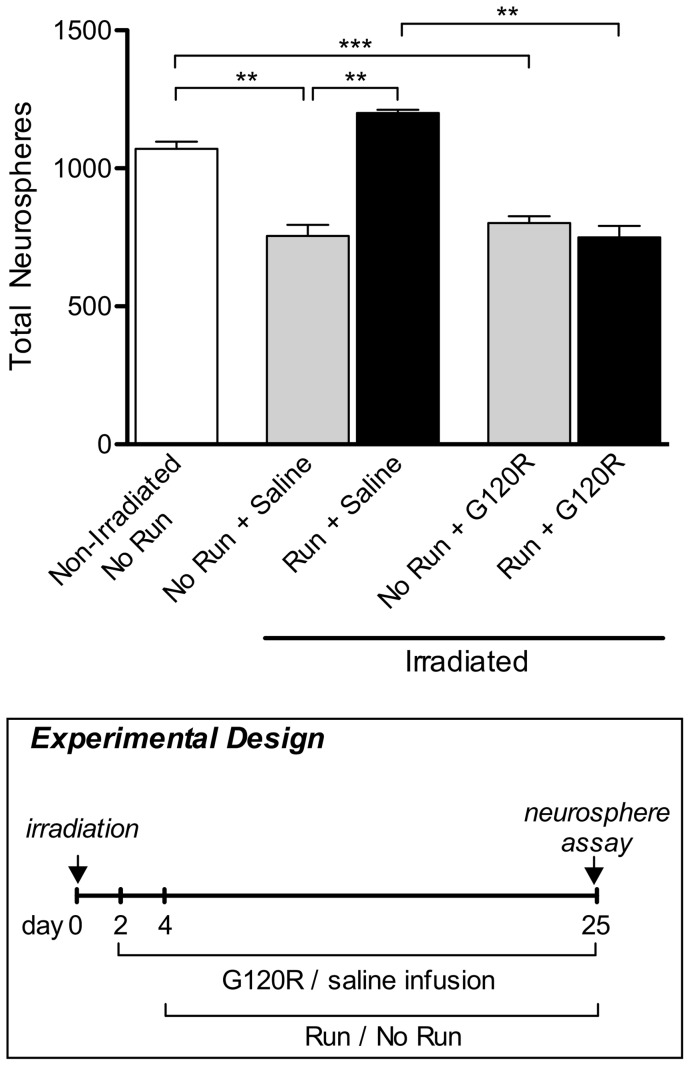
Infusion of GH antagonist during exercise prevents neural precursor cell activation in irradiated aged animals. Irradiated, sedentary animals infused with saline (“No Run + Saline”) showed significantly fewer neurospheres (p<0.01) than non-irradiated, sedentary, untreated (“Non-Irradiated, No Run”) animals. Fewer neurospheres were also observed in irradiated, sedentary animals infused with G120R (“No Run + G120R”; p<0.001). Irradiated, saline-treated animals allowed to exercise for 21 days (“Run + Saline”) produced significantly more neurospheres than the irradiated, sedentary, saline-treated animals (p<0.01). This increase in total neurosphere number was abolished after infusion, as irradiated animals infused with G120R and allowed to exercise for 21 days (“Run + G120R”) produced significantly fewer neurospheres than irradiated, exercised, saline-treated animals (p<0.01). Boxed panel shows schematic outlining experimental design. n = 3 for all experimental conditions. One-way ANOVA with Bonferroni's multiple comparison post hoc test. **p<0.01, ***p<0.001.

### Neurosphere passaging

Adult neurospheres were collected by centrifugation (5 minutes, 100 g), resuspended and incubated for 4 minutes in 0.1% trypsin-EDTA, followed by washing with trypsin inhibitor in HEM. Neurospheres were again collected via centrifugation (5 minutes, 100 g) and resuspended in 1 ml neurosphere assay medium and then mechanically dissociated to obtain single cells. Cell counts were conducted with T25 culture flasks re-seeded at a density of 2.5×10^5^ per flask. Adult neurosphere cultures were passaged every 7 days *in vitro*.

### Statistical analysis

Factorial design one-way analysis of variance (ANOVA) with Bonferroni's multiple comparison post hoc test or a Student's t-test was used to analyse data as appropriate (Prism 5, Graphpad Software Inc.). All values are expressed as mean±standard error of the mean unless otherwise indicated.

## Results

### Growth hormone mediates an exercise-dependent increase in neural precursor cell number

We first determined the relationship between exercise and GH in the aged brain using the neurosphere assay [9] as a read-out of neural precursor cell (NPC) number. In aged (12-month-old) animals, we observed a significant increase in primary neurosphere number in the SVZ of wild-type (WT) mice after 21 days of voluntary running, compared to control animals without access to a running wheel (p<0.01; Figure 1A). However, no increase in neurosphere numbers was observed in exercised compared to non-exercised GHR^−/−^ mice (Figure 1A).

In order to exclude prolactin (PRL), a peptide hormone homologous to GH, as a mediator of the exercise-induced precursor activation, we investigated the effect of exercise on a cohort of PRL null (PRL^−/−^) animals. Non-exercised PRL^−/−^ animals produced similar neurosphere numbers to non-exercised WT animals (Figure 1A). However, after exercise, the PRL^−/−^ mice showed a significant increase in total neurosphere number compared to both the non-exercised PRL^−/−^ animals (p<0.001; Figure 1A) and the exercised WT animals (p<0.01; Figure 1A). These findings suggest that PRL is not involved in any significant exercise-mediated activation of NPCs, and that the positive effects of running are being regulated by the activity of GH. We next investigated the effect of locally targeting GH signaling by infusing the competitive GH antagonist G120R into the lateral ventricle of aged WT mice during the 21-day exercise period. Saline-treated animals that were allowed to exercise showed a significant increase in neurosphere number compared to non-exercised mice (p<0.01; Figure 1B). No difference was observed between the saline-treated animals and the untreated animals after exercise (1392±43.4 versus 1305.7±39.5 neurospheres), indicating that the pump itself had no effect on neurosphere number. The infusion of G120R into the lateral ventricle of WT mice during exercise completely abolished the increase in neurosphere numbers observed in exercised WT controls (p<0.01; Figure 1B), revealing that the neurogenic effect of running was mediated by GH in these animals.

### NPCs from aged animals are responsive to GH *in vitro* and *in vivo*

Having established that a population of NPCs is activated during exercise and that this activation can be blocked by the administration of a GH antagonist, we investigated whether GH could directly activate NPCs in tissue from aged animals. The addition of GH to cultures of WT SVZ cells led to a significant increase in the total number of primary neurospheres compared to control conditions (p<0.01; Figure 2A). As expected, the addition of GH to cells from GHR^−/−^ animals had no significant effect on the total number of neurospheres generated (Figure 2A). *In vivo*, targeted infusion of GH into the lateral ventricle of aged WT mice for 7 days resulted in a significant increase in total neurosphere number compared to saline-infused animals (p<0.01; Figure 2B).

Further characterisation of the effect of GH on precursor activity showed that long-term passaging cultures of WT SVZ cells in the presence of exogenous GH resulted in a significant increase in proliferative potential (p<0.01; Figure 2C). GH activation was blocked *in vitro* by the addition of GH antagonist to GH-treated cells. Compared to control conditions, the addition of GH to WT cells resulted in a significant increase in neurosphere number (p<0.01; Figure 2D), whereas the addition of G120R alone to WT cells had no significant effect (Figure 2D). The addition of both GH and G120R to cell cultures abolished the GH-dependent increase in neurosphere number (p<0.01; Figure 2D). Consistent with the idea that GH is involved in the activation of the same NPCs as those activated during exercise, the addition of exogenous GH to cultures containing SVZ cells from exercised animals failed to produce an additive increase in neurosphere numbers (Figure 2E).

### Exercise induces activation of NPCs in the irradiated aged brain

Given that the aged brain does not recover well after injury, we sought to determine whether physical exercise could promote NPC activation in the irradiated aged brain. In a previous study, we reported that in mice older than 12 months of age, a low dose of irradiation prevented the return of NPC numbers to pre-irradiation levels [2]. In the mouse, the apoptosis that follows irradiation has been reported to peak 12 hours post-irradiation and to be completed by 48 hours [20]. Therefore, to avoid the possibility of exercise exacerbating this response, cohorts of aged WT mice were allowed to recover for 2 days after being irradiated with 3.5Gy. After the 48-hour rest period, mice were allowed to exercise for either 7 or 14 days. In both cases, the irradiated animals that had access to a running wheel showed a significant increase in neurosphere numbers compared to their more sedentary, irradiated counterparts (p<0.01; Figure 3).

### GH infusion induces activation of NPCs in the irradiated aged brain

We next investigated whether *in vivo* infusion of GH could mimic exercise-mediated NPC activation in irradiated aged animals. At 14 days after infusion, there was a significant increase in SVZ neurosphere numbers in irradiated, GH-treated animals compared to the irradiated, saline-treated control (p<0.001; Figure 4). Three days following infusion, we also observed a significant increase in neurosphere numbers in irradiated animals that had received GH compared to both the non-irradiated, untreated animals (1161±90.85 versus 863.7±67.96 neurospheres; p<0.01) and the irradiated, saline-treated animals (395.3±7.22 neurospheres; p<0.001). Thus, GH infusion repopulated the irradiated SVZ to a similar extent to exercise.

### Antagonism of GH during exercise prevents NPC activation in irradiated aged animals

Finally, we investigated whether the constant infusion of GH antagonist could block the effects of exercise following irradiation. In the presence of G120R the exercise-mediated rescue of precursor cell activity was suppressed, with neurosphere numbers remaining at a level comparable to that observed in irradiated, saline-treated, sedentary animals and irradiated, G120R-treated, sedentary animals (Figure 5). Irradiated, saline-treated animals, which were allowed to exercise for 21 days demonstrated a significant increase in SVZ neurosphere numbers compared to irradiated, saline-treated, sedentary animals (p<0.01; Figure 5). This result demonstrates that inhibition of GH activation prevents the exercise-induced recovery of NPC number within the SVZ of irradiated aged animals.

## Discussion

In the present study we have demonstrated that GH mediates the activation of NPCs within the SVZ of aged mice after exercise. We have shown that the addition of exogenous GH to WT cells derived from aged brains *in vitro* results in an increase in neurosphere numbers. Consistent with these findings, we also demonstrated that direct infusion of the GH antagonist G120R into the lateral ventricle blocks the exercise-dependent activation of SVZ NPCs. We then examined the effect of exercise post-irradiation and found that only those irradiated animals which had been provided with access to a running wheel showed NPC repopulation in the SVZ. This activation could be blocked by infusion of G120R during exercise. Following irradiation, the *in vivo* infusion of GH also resulted in significant SVZ NPC repopulation.

It has been well established that exercise results in an increase in cell proliferation and neurogenesis within the dentate gyrus of the hippocampus [4], [6]. GH has also previously been implicated, *in vitro*, in the induction of adult neurogenesis, gliogenesis and precursor cell division [14], [15], [21], [22]. Furthermore, we have previously shown that intracerebroventricular infusion of GH increases the number of newborn neurons within the adult olfactory bulb [14]. Previous reports have also demonstrated that GH-deficient rodents [23] and humans [24] have reduced cognitive function, some of which can be alleviated with exogenous GH treatment [25], [26]. In the current study we observed that the addition of exogenous GH to WT cells derived from aged SVZ preparations *in vitro* resulted in an increase in NPC numbers, and that the *in vivo* infusion of exogenous GH induced NPC activation in the aged brain, as evidenced by an increase in neurosphere number. This is a particularly important finding given that NPC numbers in both the SVZ and hippocampus progressively decline with age [2], [3] and that potential treatments to reverse the reduction in precursor activation are limited. Our data demonstrate that the SVZ of the aged brain retains a population of precursor cells that can be activated with GH and exercise.

Furthermore, our findings demonstrate a direct link between exercise and GH-induced NPC activation. Intracerebroventricular infusion of the GH antagonist G120R during exercise abolished the exercise-mediated increase in NPC activation. PRL has also been implicated in the activation of NPCs in both rodents [27], [28] and humans [16]. We excluded the involvement of PRL signalling in NPC activation following exercise, as exercised PRL^−/−^ animals displayed an increase in neurosphere number, which was higher than that of the exercised controls. This increased activation of NPCs is difficult to explain on the basis that GH has increased activity due to access to an unoccupied PRL receptor (PRLR) since in rodents, as opposed to humans, GH does not bind to the PRLR [29], [30]. Also, since PRL signals only through the PRLR and does not bind to GHR in all species examined [30], [31], it is unlikely that this increased activation is due to increased binding of GH to its receptor due to lack of competition. One explanation is that signaling through the PRLR is inhibitory, though this is unlikely as it has recently been shown that PRL is able to activate hippocampal precursor cells both in vivo and in vitro [32]. Another explanation that needs to be further examined is that this increased activation results from a rise in GH levels in the PRL deficient mice. As such, the observed exercise-mediated NPC activation appears dependent on GH signaling. Given that exercise is one of the most effective means by which to stimulate NPCs in the aged brain, our study indicates that GH is an important therapeutic target to pursue, as it can mimic the beneficial effects of exercise on precursors in the aged brain.

In a previous study we demonstrated that after irradiation, precursor cell numbers were unable to return to non-irradiated levels in the aged rodent system [2]. Other studies have previously shown that irradiation induces a significant reduction in proliferating cells [33], neurogenesis [20] and cognitive function [34], [35]. Here we have demonstrated that voluntary exercise after irradiation is robust enough to induce repopulation of the SVZ in aged animals, returning precursor numbers to pre-irradiated levels. This is in agreement with previous studies demonstrating that in young animals, voluntary exercise after irradiation rescues hippocampal neurogenesis [36] and cognitive function [37].

Importantly, we found that exercise-induced NPC recovery after irradiation could be mimicked by the direct infusion of GH. These data provide a basis for understanding previous studies of neural regeneration by GH following hypoxic-ischemic injury [38]–[40]. We postulate that in addition to being neuroprotective, as suggested by Han and colleagues [41], GH can induce the activation of the endogenous NPC pool in the injured, aged brain. Our observation that NPC numbers could not be recovered with exercise in irradiated animals infused with a GH antagonist demonstrates that, in the SVZ, GH-induced NPC activation and exercise-induced NPC activation are linked. It also demonstrates that the machinery for NPC activation remains following irradiation, allowing it to be triggered by both exercise and GH treatment.
